# Correlation of a Temperate UV-Weathering Cycle to Outdoor Exposure for the Determination of the Environmental Instability of Polyethylene Films Using HT-GPC Analysis

**DOI:** 10.3390/polym13040591

**Published:** 2021-02-16

**Authors:** Gavin Hill, Celine Moreira, Florence Huynh, Ana Trufasila, Faith Ly, Richard Lloyd, Hasan Sawal, Christopher J. Wallis

**Affiliations:** Polymateria Limited, i-Hub, Imperial College White City Campus, 84 Wood Lane, London W12 OBZ, UK; gh@polymateria.com (G.H.); cm@polymateria.com (C.M.); fh@polymateria.com (F.H.); at@polymateria.com (A.T.); fl@polymateria.com (F.L.); rl@polymateria.com (R.L.); hs@polymateria.com (H.S.)

**Keywords:** accelerated UV-weathering, polyethylene, outdoor exposure, weathering correlation, HT-GPC, carbonyl index, molecular weight

## Abstract

Accelerated UV-weathering cycles are predominately used for evaluating the durability of plastic materials, particularly polyethylene (PE) films. The point of failure for this testing is usually the loss of a physical property, such as the loss of tensile strength over time. For plastics designed to be instable under environmental conditions, the accelerated weathering cycles are yet to be defined and their correlation to outdoor exposure has yet to be made. This study demonstrates the utility of a newly defined temperate accelerated UV-weathering cycle, recently codified in the British Standard PAS 9017:2020. In addition, the effectiveness of the laboratory weathering cycle has been correlated to real-world outdoor exposure through simultaneous testing of the same samples at a specialist outdoor exposure site in Florida. The utility of the testing methodology and the performance of the polyethylene samples was demonstrated through the use of High Temperature Gel Permeation Chromatography (HT-GPC) analysis. The data led to a detailed insight into the physico-chemical changes occurring in the PE films upon exposure to environmental stimuli. By comparison, and surprisingly, the techniques employed appear to provide an insight into the processes in which secondary micro-particles of PE are formed from macro-polyethylene samples. The temperate accelerated UV-weathering cycle over 14 days demonstrated an approximate correlation to 90 days of outdoor exposure in Florida for the PE film studied.

## 1. Introduction

The stability of plastic materials is a key determining factor for many plastic products whose application is based upon outdoor use. Examples of this include, piping, automobile parts and even packaging, to name just a few. Consequently, the Service Life Prediction of these polymeric materials has gained scientific enquiry since the large-scale adoption of polymers as commercial materials [1]. It is equally well-noted that the determination of the Service Life Prediction of a polymer is dependent on the point of failure chosen, as determined by the testing methodology employed [1,2]. Hence the point of failure of a plastic bag is often chosen to be a decrease greater than 50% of its original elongation at break, as this would indicate the point at which it can no longer hold a sufficient quantity of load.

The weathering of polyolefinic materials has received significant study due to the ubiquitous use of polyolefinic materials in many outdoor applications. It therefore became critical to correlate accelerated laboratory weathering techniques to outdoor exposure [3,4]. Polyethylene and polypropylene are susceptible to degradation through oxidative-UV irradiation, often resulting in the formation of secondary polyolefinic microplastics. International standards currently govern the use of UV-accelerated weathering, namely ASTM D4329-13 [5] and ISO 4892-3 [6]. Moreover, standards governing the outdoor exposure methodologies for plastics, such as ASTM G113 [7], state that indoor accelerated tests should match results obtained through outdoor exposure. To this end, recent studies have been conducted that have shown the correlation between the durability of polypropylene in laboratory accelerated weathering compared to outdoor exposure in different regions of China [8] and Japan [9]. Furthermore, similar correlated studies have been conducted on polymers used as high-voltage insulators on overhead electric power cables for trains in India [10].

The point of failure for these studies focused upon determining the durability of these materials and thusly, the weathering cycles used in the laboratory accelerated testing are often designed to simulate multi-years’ worth of UV irradiation. Interestingly, whilst the majority of studies into laboratory accelerated testing have focused on durability, little work has been done on cycles that are specifically designed to simulate a very short outdoor exposure time. The necessity for such cycles has been gathering due to the increased awareness of plastic pollution accumulating in the natural environment, combined with the need to evaluate the production rate of secondary microplastics from primary microplastic pollution [11]. In addition, technology solutions to such a problem need to be verified as having a real-world impact in real-world environments to determine their performance, whilst evidencing their effectiveness, as recently codified in BS PAS 9017:2020 [12]. To that end this communication presents a temperate accelerated weathering cycle for UV-aging of polyolefin materials. To demonstrate the correlation to outdoor exposure, the accelerated laboratory method is compared to outdoor exposure in Florida, where outdoor exposure was conducted at a specialist site. The correlation between the performance of standard polyethylene films and polyethylene films containing Polymateria’s Biotransformation^TM^ technology is demonstrated by comparison of the loss of the polymer structure over time.

## 2. Materials and Methods

### 2.1. Materials

The samples for the accelerated and outdoor weathering study were produced on Collin Film Blowing line BL 180/400 where additive dosage can be well controlled (Collin Lab & Pilot Solutions, Maitenbeth, Germany). Sample SF 01 was a monolayer polyethylene film (87.5 µm thick) composed of 75% low density polyethylene (LDPE) (ExxonMobil LD150 BW) and 25% linear low-density polyethylene (LLDPE) (Total Lotrene 1018H). Sample SF 02 contained the proprietary biotransformation additive [13] dosed at 2% by weight (87.5 µm thick).

### 2.2. Methods

Outdoor weathering was carried out at Q Lab Corporation (Westlake, OH, USA) testing site close to Miami, Florida; following the specification of ASTM D1435 20 [14]. Samples were cut to 30 cm × 15 cm and held in high density polyethylene (HDPE) net to prevent sample cross contamination and loss due to wind during the aging process. The samples were mounted at 45° south facing, weathering data were recorded in vicinity of the test racks for every day of testing: average daily temperature, humidity, rain fall and irradiance (UV irradiance measured between 285 nm and 385 nm with Eppley TUVR 295–385 nm). Samples measured in Florida are suffixed _Fl.

Accelerated laboratory weathering testing for polyethylene (PE) film was carried out by Polymateria Ltd. using a QUV A fluorescence tester from Q Lab Corporation (Westlake, OH, USA) where irradiance and black panel temperature were calibrated at 340 nm. An irradiance setpoint of 0.8 W/m^2^ and BPT of 60 °C for 1 h with 23 h dark at 60 °C in 24 h repeated over 14 days. Samples prepared by the QUV cycle are suffixed with _QUV

Infra Red analysis was carried out on either a Nicolet iS10 or a Nicolet iS5 equipped iD7 Diamond ATR (Thermofisher Scientific, Waltham, MA, USA) between 4000 and 600 cm^−1^ at a resolution of 4 cm^−1^. The carbonyl index was calculated using the SAUB method [15] where CI = Area (1850–1650)/Area (1500–1420). This method was recommended as it accounted for the full range of carbonyl species generated during the weathering. 

Molecular weight analysis was carried out using an Agilent 1260 Infinity II High Temperature Gel Permeation Chromatography system equipped with Refractive Index detector (Agilent, Santa Clara, CA, USA), 2× Olexis PL Gel columns and calibrated using PS standards. Samples were measured at 160 °C at a flow rate of 1 ml/min and samples were dissolved and analyzed according to ASTM D6474 20 [16]. For most HT GPC experiments only a single measurement was recorded, this is due to time and cost in running samples and that the HT GPC has a high reproducibility when samples are prepared correctly.

## 3. Results and Discussion

The key to correlating the performance of a polyolefin material relative to environmental stress is the identification of the point of failure relative to the material’s application. In the case of polyolefins designed to biodegrade in the open environment, the publication of PAS 9017:2020 [12] has determined the point of failure of the polyolefin to be a significant loss in the molecular weight of the polymer, as determined by high temperature gel permeation chromatography, in addition to a significant increase in the Carbonyl Index as measured and calculated from infrared spectroscopy using the Specific Area Under Band (SAUB) technique [15]. The aim of this work was to use these analytical methodologies to demonstrate the point of failure through the loss in the chemical properties of the polyethylene matrix over time, under both types of environmental exposure—accelerated and outdoor, thus demonstrating the utility of this weathering technique as a temperate UV-accelerated laboratory method to simulate environmental instability of polyethylene materials over a relatively short timeframe. The polyethylene materials chosen were two films of 87.5 µm thick each: SF-01, a film consisting of 75% LDPE and 25% LLDPE; and SF-02 consisting of the same composition with the additional of 2% by weight of the Biotransformation^TM^ technology.

### 3.1. Outdoor Expsoure Sites

The weathering of the polyethylene (PE) samples was carried out at the Q-Labs test site in Florida (USA), which is considered a warm/humid test environment. The samples were angled 45 °C to the sun on racks. The average daily temperatures and UV irradiance was recorded (285 nm and 385 nm) over the total calendar period of 4 months of the experiment (Figure S1, from Supplementary Materials). The PE samples were exposed outdoors for a total of 90 days. One of the reasons Florida is often used is that the weathering remains fairly consistent over the exposure period, both temperature and daily UV irradiance do not change significantly over the period of exposure. During exposure in Florida received 22 days of precipitation, an amount equaling approximately 10 mm (Figure S2, Supplementary Materials).

Outdoor weathering in Florida is ubiquitously used in many industries from paints, plastics to automotive and aerospace to evaluate the effects of weather on materials and is used widely for the purpose of correlating laboratory weathering with outdoor exposure in the evaluation of plastic durability [1]. Due to the remoteness of the Florida exposure site, the average temperature experienced there was compared with the average temperatures for the same three months in the year 2020 for more recognisable global cities (Table 1). A good correlation was found for the average temperatures observed in these cities with respect to the Florida exposure site.

### 3.2. Correlation of Accelerated UV-Aging with Florida Outdoor Exposure

Durability studies on outdoor exposure of PE films often choose the loss of physical properties, such as elongation at break or changes in colour, as their point of failure. The focus of this study, however, is the environmental instability of the PE over the exposure time and thus the physico-chemical property changes are of much greater importance. It is well-known that surface changes upon the PE matrix can lead to the loss of physical properties, whereas at a molecular level the physico-chemical properties of the bulk material remain relatively unchanged. It is this difference in bulk versus surface effects that directed this study to focus on chemical analysis that would be more reflective of the changes occurring throughout the PE polymer structure rather than at the surface. The focus has, therefore, been on the changes in the molecular weight of the PE over time, a method that has been somewhat under-used, wherein many previous studies only recording values at the beginning and end of the experiments. Ly and co-workers [8] are amongst the few to extensively monitor molecular weight data throughout the outdoor exposure timeframe, which provided important insights into the durability of polypropylene. Moreover, the use of molecular weight analysis in the work of Ly et al., made it possible to infer changes in the polypropylene properties from its molecular weight and molecular weight distribution, and thus provided a more robust correlation between the methods of accelerated laboratory weathering and outdoor exposure. In this study the molecular weight analysis of the PE samples was performed using high temperature gel permeation chromatography, performed at 160 °C using trichlorobenzene as solvent.

**Figure 1:**
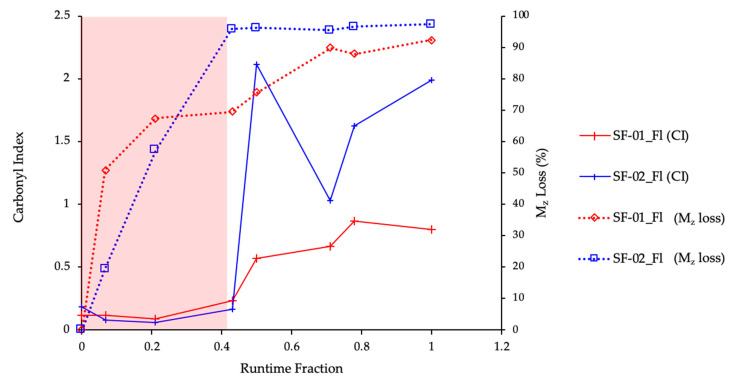
Comparison of Molecular Weight and Carbonyl Index for the PE films upon outdoor exposure in Florida.

In this study, the same PE-based films were aged in a UV-accelerated (QUV) machine for a total of 14 days. The weathering cycle was not the durability cycles as built into the machine but a temperate cycle of 1 h of UV-exposure followed by 23 h of dark. The temperature under both scenarios is held constant at 60 °C. This new cycle of testing was developed for BS PAS 9017:2020 and this communication importantly compares this laboratory UV-weathering cycle to outdoor exposure in order to verify its utility. Therefore the same samples were also aged for a total of 90 days of outdoor exposure in Florida. The effect of both types of exposure on changes to the molecular weight of the PE was monitored at regular time intervals. In order to plot and compare the results from accelerated weathering and outdoor exposure, the runtime fraction was developed (Equation (1)) to be used as a standard point of comparison, rather than the absolute time (hours or days) of exposure. With this standard timeframe measurement in place, values for number average molecular weight (M_n_), weight average molecular weight (M_w_) and Z-average molecular weight (M_z_) were recorded at regular time intervals through sacrificial sampling of the films under exposure (Tables S1 and S2, Figure 1).

(1) $$Runtime\;Fraction=\frac{Time_{n}}{Total\;time}$$

The Carbonyl Index (CI) was also measured at the same sampling intervals and calculated using the SAUB methodology [15]. As a first point of comparison, the CI data for the PE film in Florida was overlaid with the percentage (%) loss in its original M_z_ value (Figure 1.) [17]. The red shaded area shows that the majority of the losses in M_z_ occurred within the first half of the runtime fraction, whereas the increase in CI changes occurred in the second half of the runtime fraction. It is well understood that the CI changes are measured predominantly upon the surface of the material, thus while they are a strong indicator of changes in the chemical structure their interpretation should be handled with care, as without further analysis they may not reflect chemical changes occurring within the bulk of the polymer matrix. The key difference in this case, was SF-01 and SF-02 showed different trends in their physico-chemical changes upon Florida outdoor exposure. This is unsurprising given that SF-02 was designed to be instable upon environmental exposure whereas SF-01 was not. What was surprising, on the other hand, was the observed decreases in molecular weight. Whilst different in magnitude, they showed a similar trend in that they occurred in the first 50% of the total runtime and appeared to plateau afterwards. By contrast, the CI of SF-02, as expected raised sharply to a CI of approximately 2, whereas SF-01 reached a maximum CI of approximately 0.8. To that end, it is clear that measuring molecular weight losses was a more accurate and representative methodology for determining the degree of environmental instability of PE materials over time. 

An attempt was made to look at the formation of C=C double bonds (909 cm^−1^) in line with observations made by Gardette [18] and others [19], however, the bands were not sufficiently strong for most samples to allow differentiation from the baseline measurements as the materials aged (Figures S3 and S4). Only the SF-01_Fl showed the expected increase in line with Norrish photo-cleavage, however, with this sample this also corresponding to an increase in the CI (runtime fraction 0.43).

**Figure 2:**
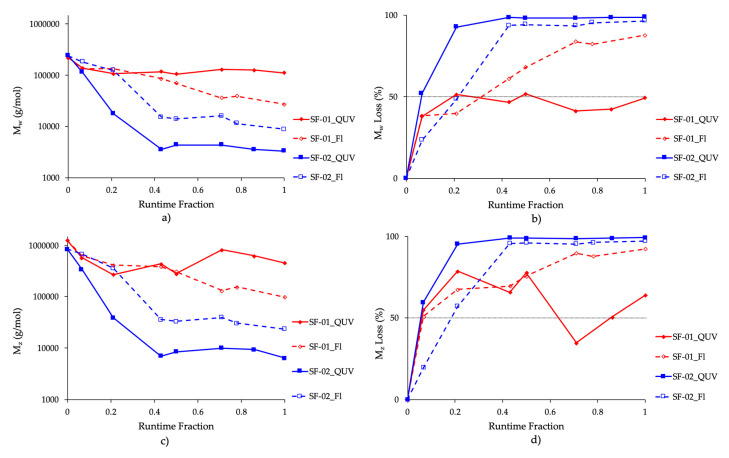
Comparison of Molecular Weight data for control and innovation samples showing: (**a**) M_w_ vs runtime fraction; (**b**) M_w_ loss vs. runtime fraction; (**c**) M_z_ vs runtime fraction and (**d**) M_z_ loss vs. runtime fraction.

The comparison in the timings of the molecular weight changes was further used to determine the correlation between outdoor exposure and the temperate UV-accelerated weathering cycle (Figure 2). The results indicated that the temperate UV-weathering cycle and the outdoor exposure of SF-02, containing the Biotransformation^TM^ technology, showed a similar molecular weight loss profile. Strikingly, SF-02 lost more than 90% of its original M_w_ and M_z_ within 0.43 of the runtime fraction. This equated to 6 days of temperate UV-weathering under accelerated laboratory conditions and 39 days of Florida outdoor exposure, respectively. This correlation was equally reflected in the comparison in the reduction of the relative values of M_n_, M_w_ and M_z_ for the same runtime fraction (Figure 2, Tables S1 and S2). From the 0.43 runtime fraction onwards the molecular weight decreases appeared to plateau under both exposure conditions reaching a maximum of greater than 95% for losses in M_w_ and M_z_ (Tables S1 and S2). These data suggested that the PE, both the LDPE and LLDPE, within the composition of this film, were equally transformed into parrafin waxes. The chemical changes resulted in the physical disintegration of the film into a fine and soft powdery wax material. Furthermore, the high level of carbonyl functionalisation of these waxes, as indicated by the CI, appeared to be a function of the chemical changes imparted by the Biotransformation^TM^ technology, resulting in the complete loss of the polymeric structure of the original PE. Further studies are ongoing to understand the reaction kinetics and mechanisms in more detail.

The control sample, SF-01, showed a similar correlation, with a greater than 50% reduction in Mw and M_z_ by the 0.2 runtime fraction, but did not reach a 90% loss in neither M_w_ nor M_z_, even after the total runtime exposure. Interestingly, the M_w_ and M_z_ molecular weight reductions under the influence of outdoor exposure, continued to a much greater extent than under the temperate UV-weathering cycle. This is partly due to the fact that in outdoor conditions, samples will undergo much greater stresses that will also impact on the degradative performance, such as changes in humidity, pollution, rainfall and greater thermal shock as materials are heated and cooled during the day and night. Despite these differences, which are not always possible to mimic in a laboratory setting, the temperate cycle chosen shows a high representative correlation to the outdoor exposure of Florida. Additionally, and in contrast to SF-02, SF-01 physically disintegrates into brittle flakes of polyethylene film pieces, rather than the powdery wax material as seen for SF-02.

The relative instability of the control samples during outdoor weathering was quite surprising. With these reductions in molecular weight, it is highly likely that this would translate into a deterioration in the physical strength of the materials. It has been postulated by Scott et al. [20] and others [21] that the degradation of PE is triggered by peroxides generated during processing, and the presence and consumption of these species over time would explain the decrease in molecular weight, leading to an eventual plateau, once the residual peroxide species have all been consumed. Furthermore, the composition of the SF-01 could also explain the observed reduction in molecular weights. SF-01 contains 25% LLDPE to 75% LDPE. The LDPE is generally hypothesised to be more environmentally instable as it more readily undergoes photodegradation relative to LLDPE [11]. Considering this, the molecular weight data for SF-01 suggested a maximum Mw loss of 88% and 56% under outdoor and temperate accelerated UV-weathering, respectively. A possible explanation for this observation would be that the LDPE portion of the material photodegraded, causing the overall film to fragment into microparticles of LDPE and LLDPE. LDPE is known to be more labile to oxidise under weathering, relative to LLDPE, due in part to greater oxygen diffusion and a high degree of branching. It is also more susceptible to cross-linking due to greater likelihood of recombination of chain ends [22]. This phenomenon was also potentially captured in the data set of SF-01 and thus may be what occurred during the temperate UV-weathering cycle, where the M_z_ increased at the 0.7 runtime fraction (Table S1). This finding suggests that while the temperate accelerated UV-weathering cycle may not be ideal for testing the durability of conventional PE plastics (i.e., designed not to break down under weathering), it has surprisingly shown the potential to be used to better understand the formation of secondary microplastics of PE formed when the original macro-PE sample was exposed to environmental stimuli over a relatively short period of time.

### 3.3. Correlation of Temperate UV-Weathering Cycle to BS PAS 9017:2020 and Florida Outdoor Exposure

The temperate UV-weathering cycle reported in this paper is in-line with the BS PAS 9017:2020 standardized testing protocol. The temperate cycle devised for UV-accelerated weathering machines is, as per the protocol, to be employed for film samples that are less than 250 µm in thickness. Similarly to this study, the protocol employs a set of criteria for molecular weight reductions in order to validate that the polyolefin sample has been chemically altered into a paraffin wax. In addition, the wax must contain a specified high level of carbonyl functionalisation. Combined, these criteria must all be met within the allocated time period of 14 days of temperate UV-accelerated weathering, namely: M_n_ < 5000 g/mol; % loss in M_w_ > 90%; M_z_ < 30,000 g/mol and a CI > 1.

A comparison of the molecular weight reduction and CI at the end points for both types of weathering exposure, for SF-02 are reported with reference to the BS PAS 9017:2020 criteria (Table 2). The data demonstrate that the molecular weight reductions followed a similar trend, whilst the CI increased more rapidly in the UV-accelerated weathering scenario. Despite the magnitude both exposures scenarios produced a CI of 1 or greater. Thus, both exposure scenarios produced carbonyl-functionalised paraffin waxes from their respectively samples of SF-02, in-line with the criteria specified in PAS 9017:2020. The results for SF-02 also showed that the unique temperate UV-accelerated weathering cycle, as specified in the PAS standard, correlated to approximately 3 months of outdoor exposure in Florida. This approximation highlights the utility of the laboratory temperate accelerated UV-weathering cycle for determining the timeframe of the designed environmentally instability of SF-02.

## 4. Conclusions

This study has shown that the temperate accelerated UV-weathering cycle used can be correlated to Florida outdoor exposure conditions. For PE films designed to be environmentally unstable, the traditional accelerated weathering cycles used for durability testing would have provided an over-exposure/irradiation of the materials and no discernable evidence for their inherent performance. The temperate accelerated UV-weathering cycle employed here, on the other hand, allowed for a relative low level of UV irradiation to be applied, allowing for an accelerated effect, whilst correlating well to a minimal outdoor exposure timeframe. This shorter timeframe is the critical defining factor in determining environmental instable relative to durability. The key difference between SF-01 and SF-02 demonstrates that the same principle for environmentally durable materials holds equally true for those designed to be environmentally instable i.e., the same point of failure must be chosen for the same types of materials, relative to their designed application. Furthermore, and surprisingly, the detailed molecular weight analysis of the PE films over time revealed the potential for the use of this weathering technique to aid in the understanding of how secondary microplastics are created via the fragmentation of macro-plastics. Further work is underway to demonstrate the correlation of this and other temperate laboratory accelerated weathering cycles to multiple different geographical outdoor exposure sites.

## Figures and Tables

**Table 1:** Comparison between the average temperature in the outdoor weathering locations and cities around the world over the same 3-month period (August–October 2020).

	Florida ^(a)^	Bangkok ^(b)^	Mumbai ^(b)^	Kuala Lumpur ^(b)^
Aug-20	29 °C	30 °C	28 °C	28 °C
Sep-20	28 °C	29 °C	28 °C	28 °C
Oct-20	28 °C	29 °C	29 °C	28 °C
Avg Temp	28 °C	29 °C	28 °C	28 °C

^(a)^ Calculated from measurements performed by Q-Labs at the outdoor weathering site. ^(b)^ Calculated from data available from the National Centers for Environmental information (https://www.ncdc.noaa.gov/ (accessed on 26 January 2021)).

**Table 2:** Summary of results in accordance with BS PAS 9017:2020 criteria at the end points temperate UV-weathering and outdoor exposure in Florida.

Sample	End Time (Days)	M_n_ (g/mol)	M_w_ Loss (%)	M_z_ (g/mol)	Carbonyl Index
SF-02_QUV	14	1181	99	6371	1.9
SF-02_Fl	90	2002	96	23,384	1

## Data Availability

The data presented in this study are available on request from the corresponding author.

## Supplementary Materials

The following are available online at https://www.mdpi.com/2073-4360/13/4/591/s1, Figure S1: Weathering data for Florida showing daily average temperatures and daily irradiance values up to 95 days., Figure S2. Data showing temperature, levels of precipitation and relative humidity. Figure S5. Q-Lab certificate for the 90-day outdoor exposure of SF-01 and SF-02 in Florida, Figure S3: Overlay of IR plots from outdoor weathering in Florida. Top Left is zoomed in C=C region, Centre Top is an enhanced view of SF-01_SF_T=39d (runtime fraction 0.43). Table S1. Table showing the results of the shrink film on the QUV weathering cycle. Table S2. Table showing the results of the shrink film samples form Outdoor Weathering in South Florida.

## References

[1] White C.C., White K.M., Pickett J.E. (2017). Service Life Prediction of Polymers and Plastics Exposed to Outdoor Weathering.

[2] Albihn P. (2006). Chapter 1—The 5-Year Accelerated Aging Project for Thermoset and Thermoplastic Elastomeric Materials: A Service Life Prediction Tool, Elastomers and Components.

[3] Qin J., Jiang J., Tao Y., Zhao S., Zeng W., Shi Y., Lu T., Guo L., Wang S., Zhang X. (2021). Sunlight Tracking and Concentrated Accelerated Weathering Test Applied in Weatherability Evaluation and Service Life Prediction of Polymeric Materials. A review. Polym. Test..

[4] Sang T., Wallis C.J., Hill G., Britovsek G.J.P. (2020). Polyethylene Terephtalate Degradation under Natural and Accelerated Weathering Conditions. Eur. Polym. J..

[5] (2020). ASTM D4329-13, Standard Practice for Fluorescent Ultraviolet (UV) Lamp Apparatus Exposure of Plastics.

[6] (2016). ISO 4892-3:2016, Plastics—Methods of Exposure to Laboratory Light Sources—Part 3: Fluorescent UV Lamps.

[7] (2020). ASTM G154-16, Standard Practice for Operating Fluorescent Ultraviolet (UV) Lamp Apparatus for Exposure of Nonmetallic Materials.

[8] Lv Y., Huang Y., Yang J., Kong M., Yang H., Zhao J., Li G. (2015). Outdoor and accelerated laboratory weathering of polypropylene: A comparison and correlation study. Polym. Degrad. Stab..

[9] Shimizu K., Tokuta Y., Oishi A., Kuriyama T., Kunioka M. (2016). Weatherability of Polypropylene by Accelerated Weathering Tests and Outdoor Exposure Tests in Japan. J. Polym..

[10] Ghosh D., Khastgir D. (2018). Degradation and Stability of Polymeric High-Voltage Insulators and Prediction of Their Service Life through Environmental and Accelerated Aging processes. ACS Omega.

[11] Grause G., Chien M.-F., Inoue C. (2020). Changes during the Weathering of Polyolefins. Polym. Degrad. Stab..

[12] (2020). PAS 9017:2020, Plastics. Biodegradation of Polyolefins in an Open-Air Terrestrial Environment Specification.

[13] Chapman G., Wallis C., Hill G. (2018). Degradable Polymer and Method of Production. Patent.

[14] (2020). ASTM D1435-20, Standard Practice for Outdoor Weathering of Plastics.

[15] Almond J., Sugumaar P., Wenzel M.N., Hill G., Wallis C. (2020). Determination of the carbonyl index of polyethylene and polypropylene using specified area under band methodology with ATR-FTIR spectroscopy. e-Polymer.

[16] (2020). ASTM D6474-20, Standard Test Method for Determining Molecular Weight Distribution and Molecular Weight Averages of Polyolefins by High Temperature Gel Permeation Chromatography.

[B17-polymers-13-00591] 17.Mz was specifically selected as an indicator of molecular weight reduction as it reflects the highest weight average molecular weights within the polymer structure, and thus, is less sensitive to minor defects or changes caused during processing but is highly representative of the bulk polymer structure. Thus a large reduction in Mz reflects a large reduction in overall polymer structure

[18] Gardette M., Perthue A., Gardette J.-L., Janecska T., Földes E., Pukánszky B. (2013). Photo- and thermal-oxidation of polyethylene: Comparison of mechanisms and influence of unsaturation content. J. Pol. Deg. Stab..

[19] Martínez-Romo A., González-Mota A., Soto-Bernal J.J., Rosales-Candelas I. (2015). Investigating the Degradability of HDPE, LDPE, PE-BIO, and PE-OXO Films under UV-B Radiation. J. Spectrosc..

[20] Grassie N., Scott G. (1985). Polymer Degradation and Stabilisation.

[21] Nechifor M., Rosu M.D., Visakh P.M. (2016). Factors Influencing the Photochemical Behavior of Multicomponent Polymeric Materials. Advanced Structural Materials.

[22] Roy P.K., Hakkarainen M., Varma I.K., Albertsson A.-C. (2011). Degradable Polyethylene: Fantasy or Reality. Environ. Sci. Technol..

