# Supplementary material for: Correlation of a Temperate UV-Weathering Cycle to Outdoor Exposure for the Determination of the Environmental Instability of Polyethylene Films Using HT-GPC Analysis
Source: Polymers (Basel). 2021 Feb 16;13(4):591. doi: 10.3390/polym13040591 (PMC7920266; doi:10.3390/polym13040591)
Supplement: Supplementary file 1 [file polymers-13-00591-s001.pdf]

polymers

MDPI

Supplementary Materials

# Correlation of a Temperate UV-Weathering Cycle to Outdoor Exposure for the Determination of the Environmental Instability of Polyethylene Films Using HT-GPC Analysis

**Gavin Hill, Celine Moreira, Florence Huynh, Ana Trufasila, Faith Ly, Richard Lloyd, Hasan Sawal and Christopher J. Wallis ***

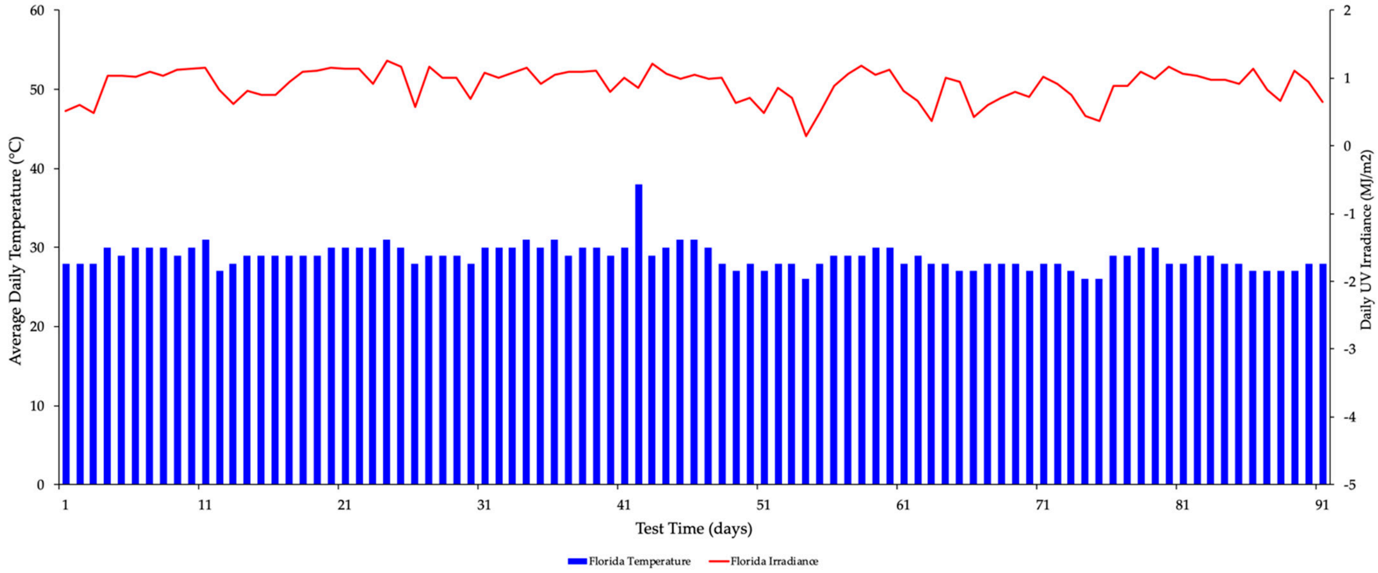


**Figure S1.** Weathering data for Florida showing daily average temperatures and daily irradiance values up to 95 days.

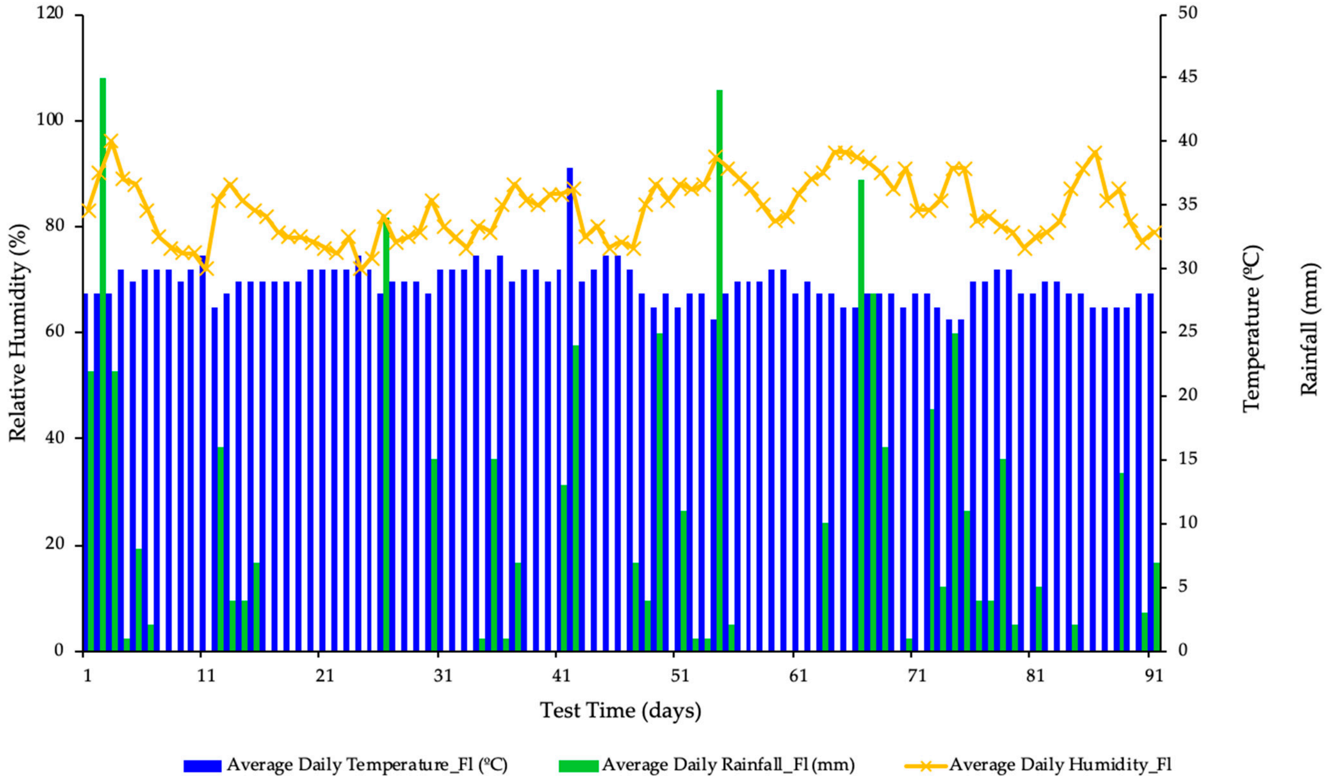


**Figure S2.** Data showing temperature, levels of precipitation and relative humidity.

**Table S1.** Table showing the results of the PE films on the QUV weathering cycle.

| | | SF-01 | | | | | SF-02 | | | | |
|---|---|---|---|---|---|---|---|---|---|---|---|
| **Time** | **Runtime Fraction** | **CI** | **Mn** | **Mw** | **Mw Loss (%)** | **Mz** | **CI** | **Mn** | **Mw** | **Mw Loss (%)** | **Mz** |
| 0 | 0 | 0.12 | 32,280 | 219,680 | 0 | 1,263,734 | 0.18 | 20,963 | 239,603 | 0 | 845,366 |
| 0.92 | 0.067 | 0.06 | 23,504 | 136,165 | 38 | 571,781 | 0.13 | 17,429 | 114,869 | 52 | 342.745 |
| 3 | 0.21 | 0.07 | 23,103 | 106,353 | 52 | 297,956 | 0.61 | 26,61 | 8,071 | 97 | 16,636 |
| 6 | 0.43 | 0.07 | 22,641 | 99,825 | 55 | 272,807 | 1.17 | 2,225 | 5,944 | 98 | 12,362 |
| 7 | 0.5 | 0.11 | 24,740 | 103,877 | 53 | 283,181 | 1.22 | 2,584 | 6,946 | 97 | 13,762 |
| 10 | 0.71 | 0.03 | 20,337 | 99,927 | 55 | 297,398 | 1.22 | 2,045 | 5,245 | 98 | 10,702 |
| 12 | 0.86 | 0.14 | 3,029 | 82,327 | 63 | 429,838 | 1.5 | 2,078 | 4,589 | 98 | 8,345 |
| 14 | 1 | 0.22 | 17,485 | 97,263 | 56 | 297389 | 2.06 | 1,745 | 4,002 | 98 | 7,589 |

**Table S2.** Table showing the results of the PE films samples form Outdoor Weathering in South Florida.

| | | SF-01_Fl | | | | | SF-02_Fl | | | | |
|---|---|---|---|---|---|---|---|---|---|---|---|
| **Time** | **Runtime Fraction** | **CI** | **Mn** | **Mw** | **Mw Loss (%)** | **Mz** | **CI** | **Mn** | **Mw** | **Mw Loss (%)** | **Mz** |
| 0 | 0 | 0.12 | 32,280 | 219,680 | 0 | 1,263,734 | 0.18 | 20,963 | 239,603 | 0 | 845,366 |
| 6 | 0.078 | 0.06 | 23,486 | 135,752 | 40 | 620,799 | 0.03 | 24,603 | 183,599 | 25 | 681,592 |

| | | | | | | | | | | | |
|---|---|---|---|---|---|---|---|---|---|---|---|
| 19 | 0.21 | 0.09 | 13,292 | 132,329 | 55 | 413,022 | 0.11 | 15,235 | 123,539 | 48 | 362,655 |
| 39 | 0.43 | 0.23 | 14,171 | 85,443 | 61 | 385,637 | 0.20 | 3,918 | 15,195 | 94 | 35,985 |
| 45 | 0.5 | 0.56 | 11,464 | 70,285 | 68 | 309,216 | 0.76 | 3,654 | 13,925 | 94 | 33,126 |
| 64 | 0.71 | 0.66 | 6,373 | 35,974 | 84 | 130,082 | 0.75 | 3,998 | 15,953 | 93 | 39,192 |
| 77 | 0.86 | 0.87 | 7,064 | 39,148 | 82 | 153,265 | 1.16 | 2,918 | 11,403 | 95 | 30,550 |
| 90 | 1 | 0.79 | 4,981 | 27,190 | 88 | 97,481 | 1.02 | 2,002 | 8,755 | 96 | 23,384 |

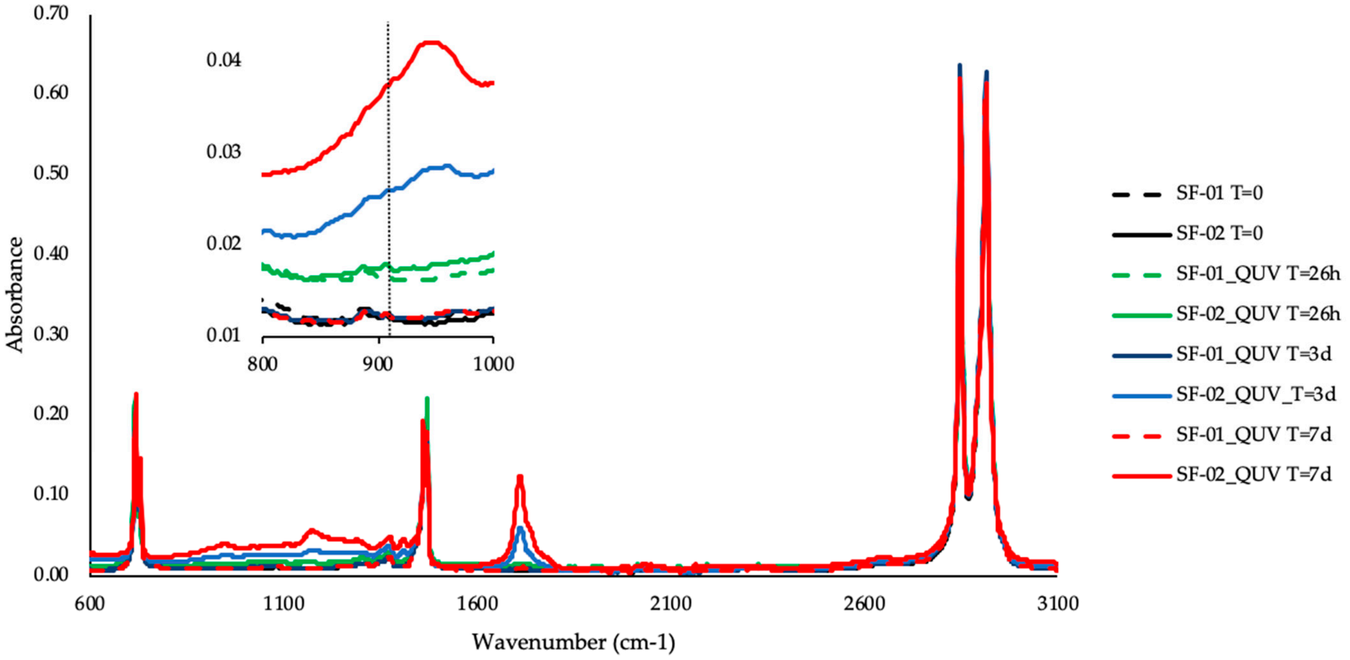


**Figure S3.** Overlay of IR plots form QUV weathering taken from first 4 Runtime Fractions. Top Left Zoomed in C=C region.

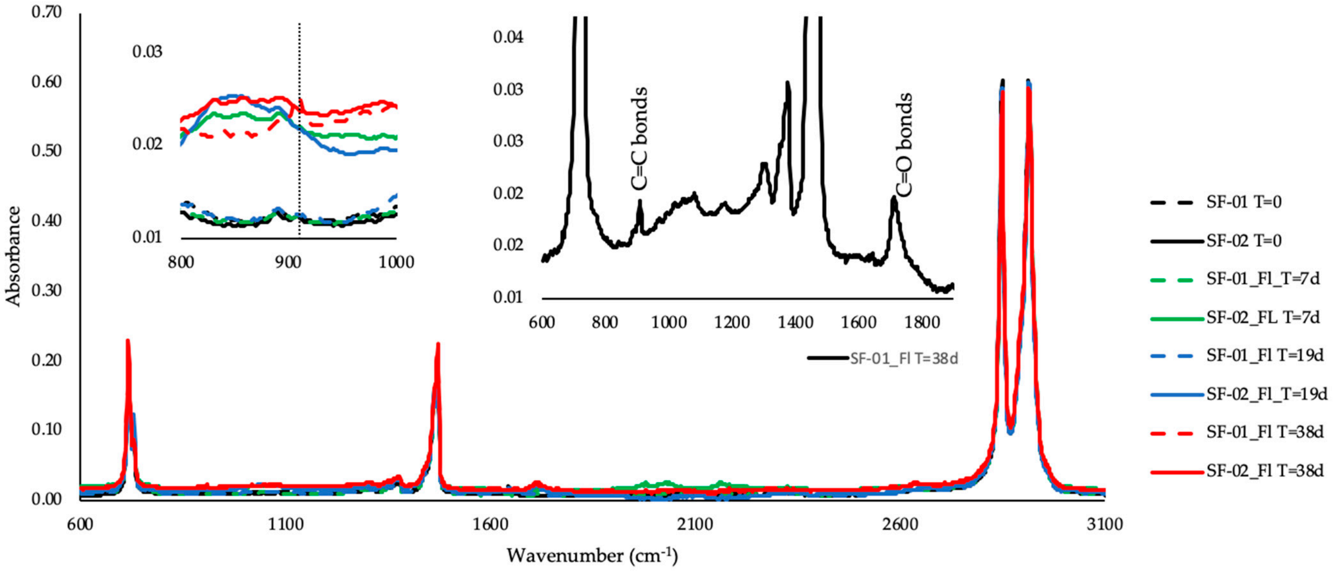


**Figure S4.** Overlay of IR plots from outdoor weathering in Florida. Top Left is zoomed in C=C region, Centre Top is an enhanced view of SF-01_SF_T=39d (runtime fraction 0.43).

Q-LAB

*Q-Lab Weathering Research Service*
*1005 S.W. 18 Avenue*
*P. O. Box 349490*
*Homestead, FL 33034*

**TEST CERTIFICATE**
Natural Weathering

| | |
|---|---|
| Test Program Number: | *PLL-4-TP-1* |
| Company: | *Polymateria Limited* |
| Address: | *Imperial College London I-HUB White City Campus 80 Wood Lane*<br>*London W12 0BZ,*<br>*UNITED KINGDOM* |
| Attention: | *Ms. Celine Moreira* |
| Your Reference: | *Project Plan 2* |
| No. Of Specimens: | *68* |
| Specimen Identification: | *See following page.* |
| Test Method: | *ASTM G7 2013* |
| Deviations: | *None* |
| Exposure Date: | *July 21, 2020* |
| Completion Date: | *October 19, 2020* |
| Exposure Duration: | *2 Months 28 Days* |
| Exposure Type: | *Direct Weathering- Florida*<br>*45° South*<br>*Backed* |
| Radiation Exposure: | *Total = 1,449.89 MJ/m2 TUVR = 81.53 MJ/m2* |

By:
Rosie Rosario
Test Set Up Supervisor

Approved By:
Michael Crewdson
General Manager

*Page 1 of 2*

**Figure S5.** Q-Lab certificate for the 90-day outdoor exposure of SF_01 and SF_02 in Florida.
